# Supplementary material for: Plasma Lipid Profiling of Three Types of Drug-Induced Liver Injury in Japanese Patients: A Preliminary Study
Source: Metabolites. 2020 Aug 31;10(9):355. doi: 10.3390/metabo10090355 (PMC7569965; doi:10.3390/metabo10090355)
Supplement: Supplementary file 1 [file metabolites-10-00355-s001.zip › Supplementary figure S1.pptx]

## Slide 1
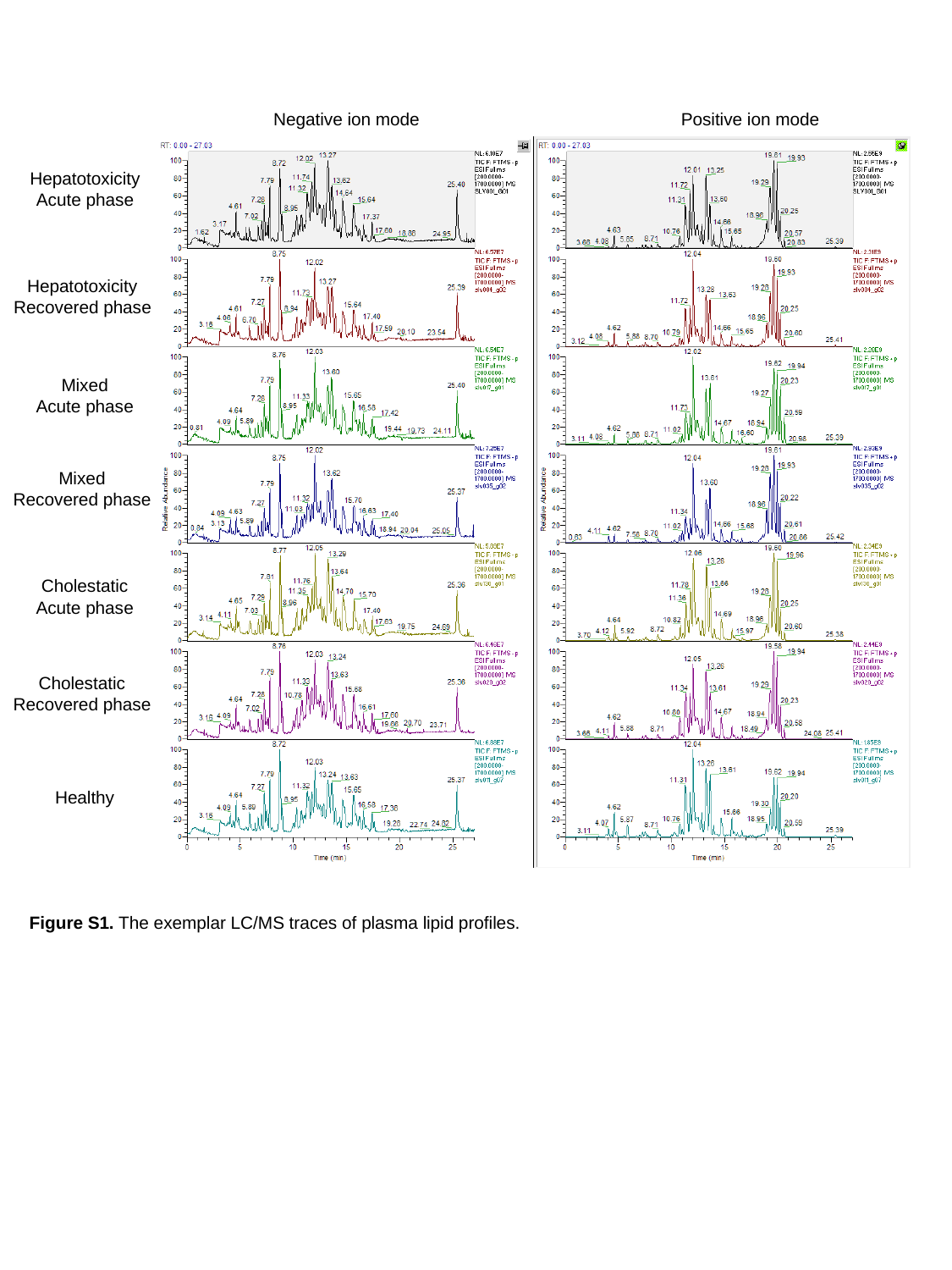

Positive ion mode
Negative ion mode
Hepatotoxicity
Acute phase
Hepatotoxicity
Recovered phase
Mixed
Acute phase
Mixed
Recovered phase
Cholestatic
Acute phase
Cholestatic
Recovered phase
Healthy
Figure S1. The exemplar LC/MS traces of plasma lipid profiles.
