# Supplementary material for: Plasma Lipid Profiling of Three Types of Drug-Induced Liver Injury in Japanese Patients: A Preliminary Study
Source: Metabolites. 2020 Aug 31;10(9):355. doi: 10.3390/metabo10090355 (PMC7569965; doi:10.3390/metabo10090355)
Supplement: Supplementary file 1 [file metabolites-10-00355-s001.zip › Supplementary text S1.docx]

Text S1. The details on the lipidomic analyses and associated data processing.

Lipid extraction was performed using the Microlab NIMBUS workstation (Hamilton, Binaduz, GR, Switzerland). The plasma samples were mixed with 9 volumes of methanol/isopropanol (1/1) containing an internal standard (PC[12:0/12:0]), which is not detectable endogenously, at 2 μM. The mixed samples were filtered through a FastRemover Protein Removal Plate (GL Science, Tokyo, Japan) using an MPE2 automated liquid handling unit (Hamilton). The resulting lipid-containing filtrate was directly subjected to LC/MS lipidomics. An InertSustainSwift C18 column (3 μm, 2.1 x 250 mm [P]; GL Science, Tokyo, Japan) was used for LC in this study. The temperatures of the column oven and sample tray were set to 55°C and 4.5°C, respectively. The mobile phase was pumped at a flow rate of 250 μl/min. Mobile phase A was composed of water/methanol/acetonitrile (21/20/60 [vol/vol/vol]) with 0.1% formic acid and 10 mM ammonium formate, and mobile phase B was composed of water/acetonitrile/isopropanol (1/10/90 [vol/vol/vol]) with 0.1% formic acid and 10 mM ammonium formate. A multistep gradient was used as follows. The gradient was initiated at 10% solvent B and subsequently increased to 40% solvent B over 5 min. The mobile phase was further increased to 50% solvent B between 5 and 10 min. The mobile phase was finally changed to 100% solvent B between 10 and 18 min before maintaining it at 100% mobile phase B for a further 4 min. The column was equilibrated with 10% mobile phase B for 5 min before the next sample was injected. After LC separation, lipids were subjected to MS using an Orbitrap Fusion instrument (ThermoFisher Scientific), operating in heated ESI mode. The spray voltage was set to 3.5 and -2.5 kV in the positive- and negative-ion modes, respectively, with the following ion-source properties: sheath gas, 45 arbitrary units (Arbs); auxiliary gas, 10 Arbs; sweep gas, 0 Arbs; ion-transfer tube temperature, 250°C; vaporizer temp, 100°C. All Fourier transform MS (FTMS) data were acquired using the following conditions: scan range, 200–1700 m/z; RF lens, 60%; automated gain control (AGC) target, 2.0e5; maximum injection time, 100 ms; microscans, 1; data type, profile. The resolving power of the Orbitrap was set at 240,000 (full width at half maximum at m/z 200). All FTMS2 and FTMS3 data were acquired using the following conditions: isolation mode, quadrupole; isolation window, 2 m/z; detector type, Orbitrap; scan range, auto; AGC target, 5.0e4; maximum injection time, 100 ms; microscans, 1; data type, centroid. Both HCD and CID were used for FTMS2 as the activation type and CID was also used for FTMS3 as the activation type. The HCD collision energy was set to 25%. The CID collision energy was set to 30% and the activation Q was set to 0.25. For lipid quantification and annotation, FTMS full scans were performed in coupled positive- and negative-ion mode. For fragment analysis, FTMS full scans coupled with FTMS2 and FTMS3 scans were performed in positive- and negative-ion mode. FTMS2 scanning was triggered by detection of ions in the inclusion list with the top 10 most intense or least intense ions having intensities greater than 1.0e4. The trigger threshold was set at 5 ppm. The ions coupled to the FTMS2 scan were excluded for 10 s until the beginning of the next FTMS2 scan. The FTMS3 scan was triggered by detection of ion loss of HCOOCH_3_ (negative-ion mode) or H_2_O (positive-ion mode) in the FTMS2 scan, with the top 3 most intense ions. Lipid ions were annotated with Compound Discover software, version 2.1 (ThermoFisher Scientific), with the threshold of lipid annotation set to 3 parts per million (ppm) variation from the expected ion mass of the major adducts and isotopic pattern. Subsequently, annotated lipids were confirmed by comparison with standards, or specific MS2/MS3 fragments and corresponding ions in the positive ion mode (only for lipids detected in negative ion mode). Our characterization identified the total carbon number, total number of double bonds, and combination of fatty acid side chains. For isomers (same class, carbon length, and number of double bonds) showing different retention times in RPLC, each lipid was assigned a metabolite ID to distinguish it. Lipids with two different fatty acid combinations (e.g., 38:6e; 18:2e/20:4, 16:1e/22:5) indicate that the quantified lipid is a mixture of two different lipids that could not be separated. The quantified raw data were normalized to the internal standard. Because the lipidomics analysis was spanned 2 batches, the median value of each lipid in all samples was set to 1 in each batch to consolidate data from 2 batches after normalization. The processed data for the lipid levels are presented in Table S1.
